# Bidirectional Interactions Between Thymocytes and Thymic Epithelial Cell Lines in Vitro

**DOI:** 10.1155/1998/80391

**Published:** 1998

**Authors:** Miodrag Čolić, Dragana Vučević, Petar Popović, Aleksandar Dujić

**Affiliations:** 1 Institute of Medical Research MMA Belgrade Serbia and Montenegro; 2 Institute of Medical Research MMA Crnotravska 17 Belgrade 11002 Serbia and Montenegro

**Keywords:** Thymic epithelial cells, thymocytes, adhesion, apoptosis, proliferation, cytokines

## Abstract

*In vitro* interactions of thymocytes and thymocyte hybridomas with cortical (R-TNC.1) and medullary (TE-R 2.5) rat thymic epithelial-cell (TEC) lines were studied. It was found that the cortical line had better adhesion capability. It bound exclusively immature CD4^+^CD8^+^ αβTCR^10^ thymocytes, induced apoptosis of a subset of these cells, and stimulhted proliferation of the BWRT (CD4^-^CD8^-^ αβTCR^-^) hybridoma. The medullary line bound both immature and mature thymocytes, decreased their apoptosis, and induced apoptosis of the BWRT 8 (CD4^+^CD8^10^ αβTCR^hi^) hybridoma. Thymocyte differently modulated cytokine production by TEC lines, upregulating the secretion of IL-1 by R-TNC.1 and IL-6 by TE-R 2.5 cells. Finally, coculture of thymocytes with TEC lines resulted in different patterns of protein-tyrosine phosphorylation in thymocytes. These results show the existence of mutual bidirectional interactions between thymocytes and TEC lines *in vitro*, but these processes differed depending on phenotypic characteristics and origin of TEC lines used.


*Developmental Immunology*, 1998, Vol. 6, pp. 71–79
Reprints available directly from the publisher
Photocopying permitted by license only

© 1998 OPA (Overseas Publishers Association)
N.V. Published by license under the
Harwood Academic Publishers imprint,
part of the Gordon and Breach Publishing Group
Printed in Malaysia

### Bidirectional Interactions between Thymocytes and Thymic Epithelial Cell Lines *In Vitro*

MIODRAG ČOLIĆ*, DRAGANA VUČEVIĆ, PETAR POPOVIĆ and ALEKSANDAR DUJIĆ

*Institute of Medical Research, MMA, Belgrade, Yugoslavia*

*(Received 5 August 1996; In final form 15 July 1997)*

*In vitro* interactions of thymocytes and thymocyte hybridomas with cortical (R-TNC.1) and medullary (TE-R 2.5) rat thymic epithelial-cell (TEC) lines were studied. It was found that the cortical line had better adhesion capability. It bound exclusively immature $CD4^{+}CD8^{+}\alpha\beta TCR^{lo}$ thymocytes, induced apoptosis of a subset of these cells, and stimulated proliferation of the BWRT 1 ($CD4^{-}CD8^{-}\alpha\beta TCR^{-}$) hybridoma. The medullary line bound both immature and mature thymocytes, decreased their apoptosis, and induced apoptosis of the BWRT 8 ($CD4^{+}CD8^{lo}\alpha\beta TCR^{hi}$) hybridoma. Thymocyte differently modulated cytokine production by TEC lines, upregulating the secretion of IL-1 by R-TNC.1 and IL-6 by TE-R 2.5 cells. Finally, coculture of thymocytes with TEC lines resulted in different patterns of protein-tyrosine phosphorylation in thymocytes. These results show the existence of mutual bidirectional interactions between thymocytes and TEC lines *in vitro*, but these processes differed depending on phenotypic characteristics and origin of TEC lines used.

*Keywords:* Thymic epithelial cells, thymocytes, adhesion, apoptosis, proliferation, cytokines

### INTRODUCTION

Thymic epithelial cells (TEC) form a supporting network of the thymus and represent the major component of the thymic microenvironment (van Ewijk, 1988). They are of crucial importance in T-cell development acting through direct cell-cell contacts with thymocytes or by production of soluble factors such as cytokines, thymic hormones, neuropeptides, and other biologically active substances (Boyd et al., 1993; Anderson et al., 1996). Thymocyte/TEC interaction is also a two-way process in which the development and functioning of TEC is dependent on the influence of developing thymocytes (Ritter and Boyd, 1993). However, little is known of the role of individual TEC subsets at different stages of T-cell development and about how these bidirectional interactions are regulated at different levels of the thymic microenvironment.

One approach to resolving this problem has been selective isolation and cultivation of individual TEC subsets *in vitro* that could reflect their normal

*Corresponding author. Pressent address: Institute of Medical Research, MMA, Crnotravska 17, 11002 Belgrade, Yugoslavia.

TABLE I Binding of Different T-Cell Populations to TEC lines

| T-cell subpopulation | Percentages of binding R-TNC.1 | TE-R 2.5 |
|---|---|---|
| Adult thymocytes | 40.3 ± 6.2 | 18.3 ± 5.3 |
| Fetal thymocytes | 53.4 ± 5.0 | 24.1 ± 3.7 |
| Neonatal thymocytes | 26.4 ± 3.9 | 16.2 ± 5.0 |
| Cortison-resistant thymocytes | 20.2 ± 4.6 | 21.3 ± 7.2 |
| Activated thymocytes (ConA + IL2) | 61.3 ± 5.9 | 39.2 ± 4.3 |
| Activated thymocytes (PMA) | 53.3 ± 5.4 | 30.2 ± 6.4 |
| Peripheral T cells | 13.2 ± 4.3 | 8.0 ± 3.4 |
| BWRT 2 hybridoma | 69.2 ± 4.6 | 29.2 ± 3.7 |
| BWRT 3 hybridoma | 79.3 ± 8.0 | 36.2 ± 7.3 |
| Adult thymocytes (IFN-$\gamma$ stim. TEC) | 58.2 ± 2.7 | 35.1 ± 5.0 |

Adhesion assay was performed as described in Materials and Methods. The percentages of bound thymocytes were determined as the mean of quadriplicates of a representative of three similar experiments.

counterpart *in vivo* and their use in various coculture experiments with thymocytes. Using such a system, we successfully cultivated and cloned two TEC lines that are of cortical and medullary origin, respectively (Čolić et al., 1991). The results presented in this work show that these TEC lines differently bind and activate thymocytes. On the other hand, thymocytes differently activate TEC, suggesting that such distinct bidirectional interactions might be relevant for the *in vivo* thymus.

### RESULTS AND DISCUSSION

### Cortical and Medullary TEC Lines Differently Bind Thymocytes and Thymocyte Hybridomas

In our institute, we established two rat TEC lines, R-TNC.1 (Čolić et al., 1994a) and TE-R 2.5 (Čolić et al., 1992), which represent subsets of cortical and medullary TEC, respectively. In this work, we demonstrated (Table I) that both lines bind thymocytes, but poorly peripheral T cells. Binding was higher when fetal or activated (PMA or ConA + IL-2) thymocytes were used in comparison to resting, adult thymocytes. However, the cortical line, which is a type of the thymic nurse-cell (TNC) line (Čolić et al., 1994a), had much higher adhesion capability. In addition, R-TNC.1 bound significantly less neonatal and hydrocortisone-resistant thymocytes. In contrast, the TE-R 2.5 line bound these cells as equally as adult thymocytes.

Phenotypic analysis (Figure 1) demonstrated that R-TNC.1 cells bound exclusively double-positive (DP) ($CD4^+CD8^+$) cells with low expression of $\alpha\beta$TCR. The finding might be relevant for the *in vivo* situation because $CD4^+CD8^+TCR^{lo}$ thymocytes are the predominant population of immature, cortical thymocytes (reviewed in Anderson et al., 1996). This is also in agreement with the strong attachment of BWRT 2 and BWRT 3 thymocyte hybridomas of cortical phenotype (Popović et al., 1994) (Table I) to the line. Similar results were published on the phenotype of intra-TNC thymocytes in freshly isolated murine TNC (Wekerle and Ketelsen, 1980) or thymocytes engulfed by mouse TNC lines (Itoh et al., 1988; Hiramine et al., 1990).

TE-R 2.5 cells also predominantly bound $CD4^+CD8^+$ thymocytes, but minor subsets of other single-positive (SP) thymocyte subsets ($CD4^+CD8^-$ and $CD4^-CD8^+$) were also identified. Based on the profile of $\alpha\beta$TCR expression, it can be concluded that among adherent thymocytes, a higher percentage of more mature ($\alpha\beta TCR^{hi}$) thymocytes was seen in comparison to those adhering to the cortical line (Figure 1).

The binding of cortical phenotype thymocytes to the medullary TEC line is not an unexpected phenomenon since similar results have already been published for a mouse medullary TEC line (Potworowski et al.,

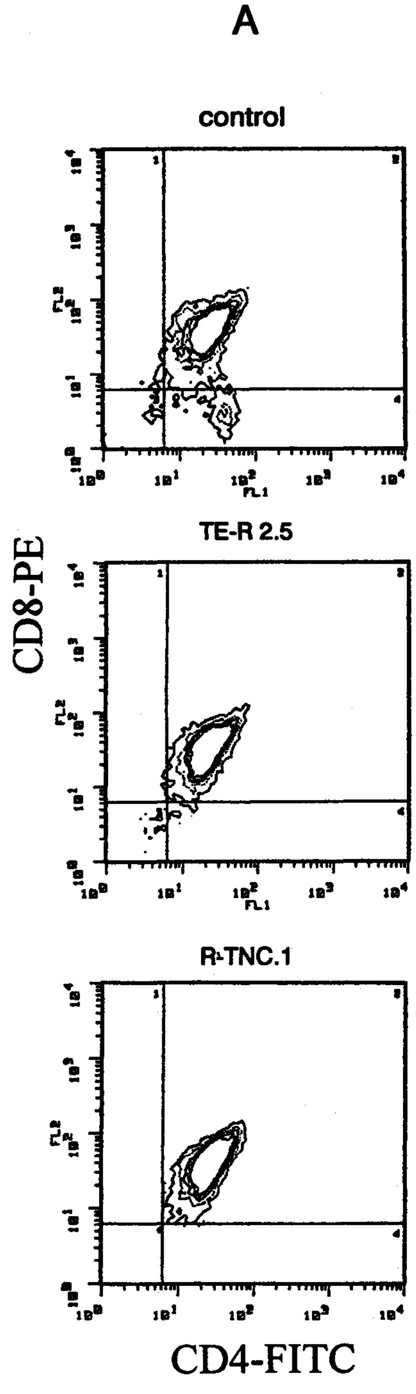

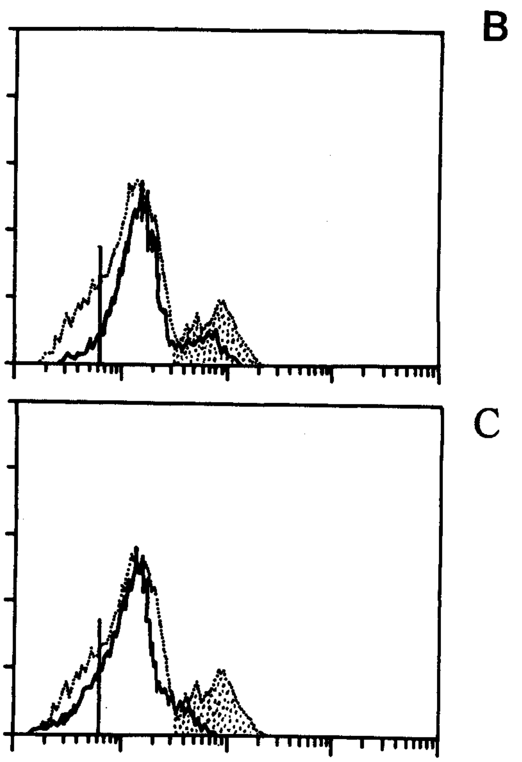

| | THYMOCYTE SUBSETS (%) | | | | | | |
|---|---|---|---|---|---|---|---|
| | $CD4^-CD8^-$ | $CD4^+CD8^+$ | $CD4^+CD8^-$ | $CD4^-CD8^+$ | $TCR^+$ | $TCR^{lo}$ | $TCR^{hi}$ |
| control | 1.60 | 87.07 | 8.18 | 3.15 | 88.26 | 72.06 | 16.20 |
| TE-R 2.5 | 1.06 | 94.20 | 2.33 | 2.41 | 86.58 | 78.03 | 8.35 |
| R-TNC.1 | 0.30 | 98.75 | 0.41 | 0.54 | 80.25 | 76.31 | 3.94 |

FIGURE 1 Phenotypical characteristics of thymocytes bound to TEC lines. Thymocytes bound to TEC lines and control, total thymocytes were stained in suspension by mAbs and analyzed on a FACScan flow cytometer. (A) Double immunofluorescence staining of control thymocytes or thymocytes that bind to the TE-R 2.5 line and the R-TNC.1 line by anti-CD4 and anti-CD8 mAbs. (B,C) Single immunofluorescence staining of thymocytes by an anti-$\alpha\beta$ TCR mAb (R-73). Solid lines represent histograms of thymocytes bound to (B) TE-R 2.5 cells and (C) R-TNC.1 cells. Dotted lines represent histograms of total thymocytes. Dashed areas represents the population of $TCR^{hi}$ thymocytes. Values represent the percentages of thymocyte subsets.

1989). We think that such a process might be relevant for *in vivo* interactions since at the corticomedullary border, medullary TEC are in close contact with immature thymocytes. In addition, our immunohistological observations in AO rats revealed that 10-20% of thymocytes located in the medulla are $CD4^{+}CD8^{+}$ (Čolić, unpublished observations).

The data presented in Table I also show that stimulation of thymocytes with PMA or TEC lines with IFN-gamma increased the adhesion process. It is known that PMA, a potent stimulator of PKC, transiently increases the affinity of LFA-1 for its ligands by inducing conformational changes of the integrin (Arnaout, 1990). We also demonstrated that PMA stimulated thymocyte binding to TEC lines via LFA-1 (Čolić et al., 1994a; Vučević et al., manuscript in preparation). IFN-gamma has been known to modulate the expression of different adhesion molecules on TEC in culture (Berrih et al., 1985). In our experiments, this cytokine upregulated the expression of class I MHC on both lines and induced the expression of class II MHC molecules and ICAM-1 (Čolić et al., 1992, 1994a). We also showed in antibody blocking studies that some of these findings, especially the expression of ICAM-1, could be relevant for increased thymocyte binding to these TEC lines (Čolić et al., 1994a).

### Cortical and Medullary TEC Lines Differently Modulate Proliferation of Thymocyte Hybridomas

We next studied the effect of TEC lines on thymocyte hybridoma proliferation. The results presented in Table II show that these lines differently modulate proliferation of phenotypically different thymocyte hybridomas. The R-TNC.1 cell line stimulated proliferation of the BWRT 1 ($CD4^{-}CD8^{-}\alpha\beta TCR^{-}$) hybridoma, whereas the growth of other hybridomas was not significantly affected. The stimulatory effect seems to be dependent on soluble factors since enhanced proliferation of the hybridoma was also observed using the R-TNC.1 supernatant.

In contrast, the medullary line, TE-R 2.5, strongly decreased proliferation of the BWRT 8 ($CD4^{hi}CD8^{lo}\alpha\beta TCR^{hi}$), which was predominantly dependent on direct cell-cell contact. Enhanced proliferation of BWRT 1 could be caused by cytokines. Among them, IL-7 is a possible candidate since of the several cytokines tested (IL-1, IL-2, IL-6, IL-7, and TNF-$\alpha$), only IL-7 had a similar stimulatory effect on the hybridoma proliferation (data not shown). It has been demonstrated that IL-7 is a growth factor for DN thymocytes and that TEC produce this cytokine (Murray et al., 1989). The stimulatory effect could be also caused by a factor produced by TEC, named TSLP (thymic stromal-derived lymphopoietin), which shares many effects with IL-7, but is structurally different (Ray et al., 1996). A definitive answer to this question will be given when genetic probes and monoclonal antibodies to these molecules in rat are available.

TABLE II Effects of TEC Lines on Thymocyte Hybridoma Proliferation

| Coculture conditions | cpm |
|---|---|
| BWRT 1 | 46,300 ± 3700 |
| BWRT 1 + R-TNC.1 | 59,200 ± 3100[a] |
| BWRT 1 + R-TNC.1 sup. | 55,600 ± 2800[a] |
| BWRT 1 + TE-R 2.5 | 42,800 ± 4600 |
| BWRT 1 + TE-R 2.5 sup. | 43,200 ± 3200 |
| BWRT 2 | 61,500 ± 4200 |
| BWRT 2 + R-TNC.1 | 63,100 ± 4200 |
| BWRT 2 + R-TNC.1 sup. | 64,200 ± 3600 |
| BWRT 2 + TE-R 2.5 | 58,200 ± 3500 |
| BWRT 2 + TE-R 2.5 sup. | 58,000 ± 5300 |
| BWRT 3 | 39,000 ± 2700 |
| BWRT 3 + R-TNC.1 | 37,400 ± 1300 |
| BWRT 3 + R-TNC.1 sup. | 41,400 ± 4300 |
| BWRT 3 + TE-R 2.5 | 35,800 ± 4600 |
| BWRT 3 + TE-R 2.5 sup. | 37,600 ± 3700 |
| BWRT 8 | 13,200 ± 1100 |
| BWRT 8 + R-TNC.1 | 12,200 ± 850 |
| BWRT 8 + R-TNC.1 sup. | 11,900 ± 1400 |
| BWRT 8 + TE-R 2.5 | 1,600 ± 300[b] |
| BWRT 8 + TE-R 2.5 sup. | 11,800 ± 2400 |

Different hybridomas ($2.5 \times 10^{3}$ well) were cultivated on mytomicin C-treated TEC monolayers or with 30% TEC supernatants as described in Materials and Methods. Thymocyte proliferation was measured by $^{3}H$ thymidine uptake and expressed as cpm ± SD of triplicate cultures. Values are from a representative experiment.

[a] $p < 0.05$ compared to corresponding controls (hybridoma alone).
[b] $p < 0.001$ compared to corresponding controls (hybridoma alone).

### Effect of TEC Lines on Apoptosis of Thymocyte and Thymocyte Hybridomas

In further experiments, we confirmed that the inhibited growth of BWRT 8 by the TE-R 2.5 cell line was a consequence of induced apoptosis of the hybridoma (Table III). The mechanisms involved in this process differed from apoptosis induced by TCR mAb cross-linking or dexamethasone treatment (Popović et al., in press). The cortical line did not induce apoptosis of the BWRT 8 hybridoma. In contrast, this line potentiated apoptosis of thymocytes (Table III). The effect was more visible after 8 hr of cell incubation when spontaneous apoptosis of thymocytes was less pronounced. Similar results have already been published for mouse TNC lines that selectively bind and eliminate (by apoptosis) DP thymocytes (Hiramine et al., 1990; Nakashima et al., 1990). Recent experiments (Aguilar et al., 1994) demonstrated that the predominant function of TNC *in vivo* could be induction of apoptosis or elimination of nonselected thymocytes. Our results (Vučević et al., unpublished) showed that the R-TNC.1 line has a dual role *in vitro*: induction of apoptosis of a subset of adherent thymocytes and stimulation of proliferation of a subset of engulfed thymocytes. These data could be related to the known function of cortical TEC in selection processes of thymocytes.

TABLE III Effects of TEC Lines on Apoptosis of Thymocytes and Thymocyte Hybridomas

| Cells | % apoptosis |
|---|---|
| Thymocytes (8 hr) | 16 ± 3 |
| Thymocytes + R-TNC.1 (8 hr) | 27 ± 4[a] |
| Thymocytes + TE-R 2.5 (8 hr) | 9 ± 3[a] |
| Thymocytes (20 hr) | 31 ± 4 |
| Thymocytes + R-TNC.1 (20 hr) | 37 ± 4 |
| Thymocytes + TE-R 2.5 (20 hr) | 22 ± 3[a] |
| BWRT 1 (20 hr) | 2 ± 1 |
| BWRT 1 + R-TNC.1 (20 hr) | 3 ± 2 |
| BWRT 1 + TE-R 2.5 (20 hr) | 5 ± 2 |
| BWRT 8 (20 hr) | 6 ± 2 |
| BWRT 8 + R-TNC.1 (20 hr) | 11 ± 4 |
| BWRT 8 + TE-R 2.5 (20 hr) | 68 ± 7[b] |

Percentages of apoptotic cells are given as mean ± SD of quadriplicates of a representative (out of three similar) experiment.

[a]$p < 0.05$ compared to corresponding controls (thymocytes or hybridomas cultivated alone).

[b]$p < 0.001$ compared to corresponding controls (thymocytes or hybridomas cultivated alone).

In contrast to these results, the TE-R 2.5 cell line decreased thymocyte apoptosis *in vitro* (Table III). A similar effect was seen when purified DP thymocytes were used (not shown). At the moment, we do not know about the mechanisms involved in the process and whether it is relevant for selection processes *in vivo*; therefore, the phenomenon is attractive for further studies.

### Thymocytes Differently Regulate IL-1 and IL-6 Production by TEC Lines in Culture

As previously found (Čolić et al., 1991, 1994b), both TEC lines used in this study spontaneously produce low to moderate levels of IL-1 and IL-6. In this work, we examined cytokine production by the lines after their cultivation with thymocytes. Figure 2 shows that after 24 hr in culture, thymocytes differently influenced the cytokine production by TEC lines. Namely, thymocytes upregulated the production of IL-1 by R-TNC.1 and IL-6 by TE-R 2.5, respectively. The reason for the opposite effect is not clear, but this can be attributed to the different requirement for IL-1 and IL-6 production during T-cell development. The production of IL-1 by TE-R 2.5 and IL-6 by R-TNC.1 was not significantly changed. It was demonstrated that both IL-1 and IL-6 enhanced the proliferation of whole unseparated thymocytes in the presence of IL-2, whereas none of them induced thymocyte proliferation alone. IL-1 enhanced the proliferation of DN thymocytes more efficiently than IL-6 in the presence of IL-2, whereas IL-6 enhanced the responses of SP thymocytes to IL-2 better than IL-1 (Suda et al., 1990). Based on these experiments, it can be hypothesized that the contact of thymocytes with the cortical TEC line enhanced the production of IL-1, which is necessary for proliferation of DN thymocytes. Both DN thymocytes and TNC are localized predominantly in the outer cortex (Wekerle and Ketelsen, 1980). In contrast, the TE-R 2.5 line is derived from a subset of medullary TEC that are in close contact with SP, more mature thymocytes. As cited before, IL-6 is a growth factor for these thymocyte subsets.

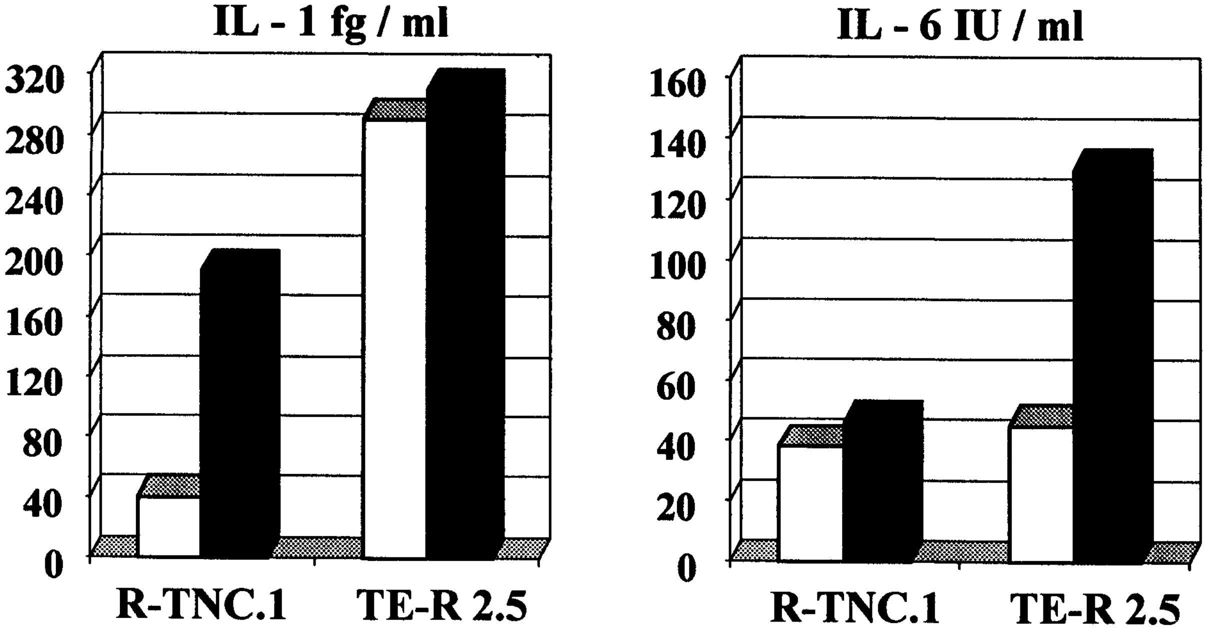

FIGURE 2 Thymocytes differently regulate IL-1 and IL-6 production by TEC lines *in vitro*. Cytokine activities were determined using sensitive D10S (IL-1) and B9 (IL-6) cell-line assays as previously described (Čolić et al. 1991). Recombinant mouse cytokines (Genzyme) were used as standards. White columns represent cytokine activities in supernatants of TEC lines cultivated alone, whereas black columns represent cytokine activities in supernatants of TEC lines cultivated with thymocytes.

It would be of interest to define signaling mechanisms involved in cytokine production by these TEC lines. One paper dealing with this problem showed that thymocytes stimulated IL-1 production by uncloned human TEC (phenotypic characteristics of TEC were not presented) through the CD2 (thymocytes)-LFA-3 (TEC) adhesion pathway (Le et al., 1990).

### TEC Lines Differently Alter Tyrosine Phosphorylation in Thymocytes

Finally, we studied tyrosine phosphorylation in thymocytes after coculture with TEC lines. Figure 3 shows different patterns of phosphorylation of a number of cellular substrates on tyrosines in thymocytes. The dominant finding was the appearance of two phosphotyrosine proteins of 110 and 120 kD at 40 min in culture of thymocytes with TE-R 2.5 but not R-TNC.1 cells. In addition, hyper- or dephosphorylation of a number of other substrates can be observed, but kinetics of the processes differed depending on TEC lines used. At the moment, we do not know the identity of these molecules. The 110-kD protein could correspond to the catalytic subunit of phosphatidylinositol-3 kinase (p110), an enzyme that has an important role in signal transduction cascade in T cells (Ward et al., 1996). The 120-kD protein has a similar molecular mass as pp125$^{FAK}$ (Mc Cormic, 1993) or rasGAP (Schaller and Parsons, 1993), which are both involved in intracellular signaling and become phosphorylated on tyrosine after activation of many cells. Thymocytes altered protein tyrosine phosphorylation in TEC lines, too (data not shown), but we did not find any *de novo* tyrosine phosphorylated substrates. The next experiments that are planned will aim to identify phosphorylated products using kinase-specific mAbs.

### MATERIALS AND METHODS

### Cells

R-TNC.1 and TE-R 2.5 cell lines were established as previously described (Čolić et al., 1991). They were cultivated using RPMI medium with addition of 15% inactivated FCS, EGF, and insulin. All ingredients were obtained from Sigma. BWRT 1, BWRT 2, and BWRT 3 thymocyte hybridomas were produced by fusing resting adult rat thymocytes with a mouse thymoma cell line (BW5147) as described (Popović et al., 1994). BWRT 8 was selected from a fusion of BW5147 cells and ConA +IL-2-activated rat thymocytes (Popović et al., 1994). All cultures were maintained at 37°C in an incubator with 5% $CO_2$. Suspensions of thymocytes were prepared from adult (10-week-old), neonatal, or fetal (17-day-old) AO rats. Thymocytes from adult rats were stimulated with PMA (20 ng/ml) for 30 min or with ConA (1 $\mu$g/ml) +3 U/ml of human recombinant IL-2) (Genzyme) for 3 days. Cortisone-resistant thymocytes were obtained after treatment of adult rats with 150 mg/kg of hydrocortisone 2 days before their sacrifice. Peripheral T cells were obtained from rat axillar lymph nodes using a nylon wool column. The purity of T cells checked by flow cytometry (R-73 mAb) was 94-97%.

### Adhesion Assay

Confluent monolayers of TEC lines grown in 96-well plates were incubated with 5 × $10^5$ thymocytes or 1 × $10^5$ hybridomas or ConA + IL-2-activated thymocytes for 1 hr at 37°C. In some experiments, TEC lines were stimulated for 2 days with 100 U/ml of recombinant

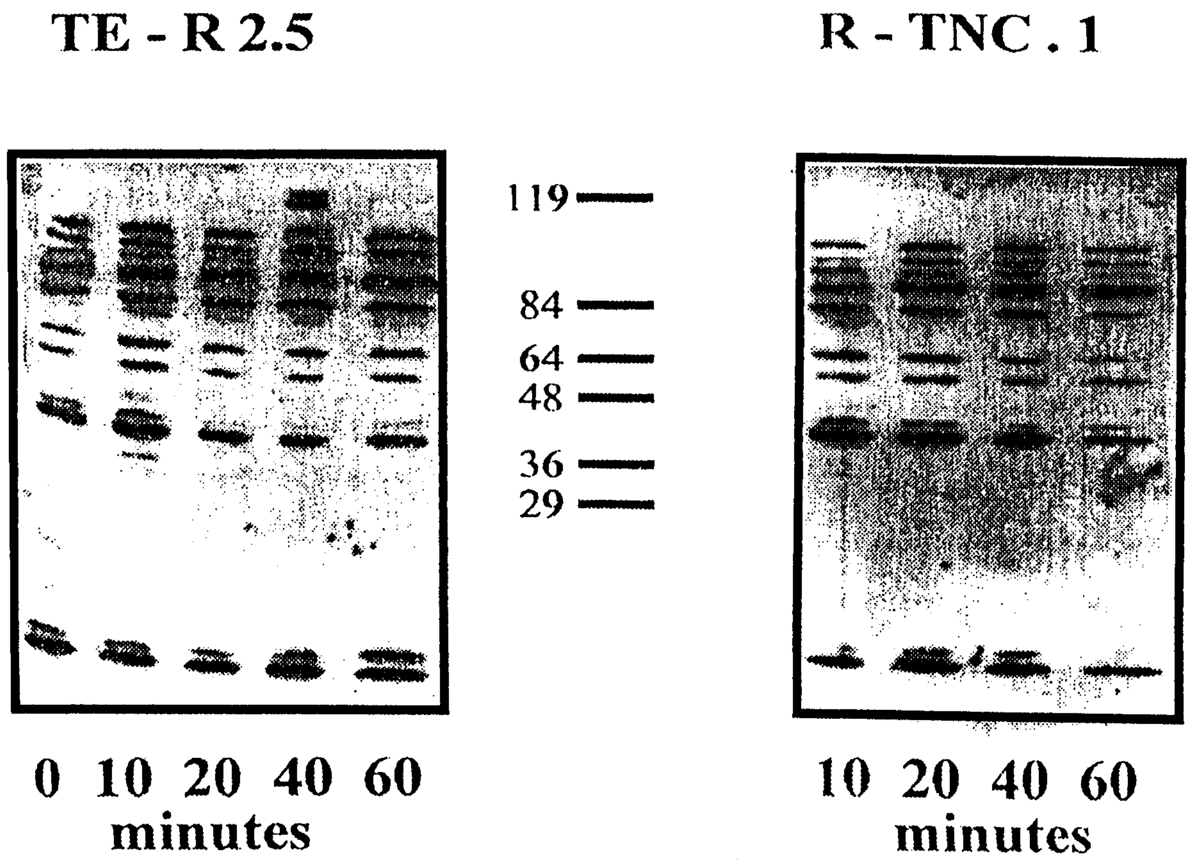

FIGURE 3 Effect of TEC lines on protein tyrosine phosphorylation in thymocytes. Thymocytes were cultivated on TEC monolayers for indicated times. Lysates were than prepared as described in Materials and Methods. Detection of proteins phosphorylated on tyrosines were determined using PY 20 mAb (Western blot). Molecular-weight standards are given in kD. Note *de novo* phosphorylated proteins of 110 and 120 kD in thymocytes cultivated with the TE-R 2.5 cell line.

rat IFN-gamma (a gift from P. van der Meide, TNO, Rijswik, The Netherlands). After incubation, nonadherent cells were discarded by gentle washing and adherent cells detached from the monolayers were calculated and expressed as percentages of bound thymocytes.

### Flow Cytometry

Adult thymocytes were bound to TEC monolayers and control; total thymocytes were stained in suspension with R-73 (anti-rat $\alpha\beta$TCR mAb) (Serotec) and secondary sheep anti-mouse Ig-FITC antibody. For double staining, thymocytes were stained with anti-rat CD4-FITC (W3/25) and anti-rat CD8-PE (OX-8) mAbs (Serotec). Controls consisted of unlabeled (single staining) or labeled (double staining) mouse Ig. Labeled cells were analyzed on a FACScan flow cytometer (Becton-Dickinson).

Values represent the percentages of cell subsets in total thymocytes population (control) or adherent thymocytes bound to TE-R 2.5 and R-TNC.1 cell lines, respectively.

### Proliferation Assay

Thymocyte hybridomas (2.5 × $10^3$/well) were cultivated on confluent TEC monolayers or with 30% TEC supernatants in 96-well plates for 48 hr at 37°C. Before addition of the hybridomas, TEC monolayers were treated with mitomycin C (Sigma) to arrest their proliferation. Hybridoma proliferation was measured after an 18-hr pulse with $^3$H thymidine (Amersham). Results are presented as the cpm of triplicate cultures.

### Apoptosis

Thymocytes (1 × $10^6$/well) or thymocyte hybridomas (5 × $10^4$/well) were incubated alone or on confluent TEC monolayers in 96-well plates for 8 or 20 hr. Apoptosis was determined after cell fixation in 4% formaldehyde in ethanol overnight and staining with hematoxylin. The percentage of apoptotic cells (cells with condensed chromatin, pyknotic, or fragmented nuclei) was determined after calculation of at least 400 cells using light microscopy.

### Cytokine Assays

The supernatant of confluent TEC monolayers cultivated alone or with thymocytes (1 × $10^6$/well) for 24 hr were tested for IL-1 and IL-6 activities using proliferation of specific cell lines D10S and B9, respectively, as previously described (Čolić et al., 1991). No cytokine activity was detected in samples of thymocytes cultivated alone.

### Phosphotyrosyne Analysis

Thymocytes (2 × $10^7$/well) were cultivated on confluent TEC monolayers in 4-well plates for indicated times at 37°C. After that, thymocytes were removed from TEC monolayers and solubilized using ice-cold TNT buffer containing 1% TritonX-100, 150 mM NaCl, 50 nM TRIS (pH 7.5), 1 mM sodium orthovanadate (ICN), and protease inhibitors obtained from Sigma (leupeptine, aprotonin, iodoacetamide, and PMSF, all at 10 $\mu$g/ml) for 15 min. Insoluble material and nuclei were removed with a 10-min microcentrifuge spin (12,000 g) at 4°C. Supernatants were added to SDS-PAGE sample buffer, boiled for 5 min, resolved on 10% SDS-PAGE, and transferred to the PVDF membrane. Detection of phosphotyrosine-containing proteins was accomplished using a mouse monoclonal anti-phosphotyrosine antibody (PY20, ICN) followed by a peroxidase-conjugated secondary antibody (DAKO). Visualization was performed using an enhanced chemiluminescence assay kit (Amersham).

### *References*

Aguilar L.K., Aguilar-Cordova E., Cartwright J., and Belmont J.W. (1994). Thymic nurse cells are sites of thymocyte apoptosis. J. Immunol. **152**: 2645-2651.

Anderson G., Moore N.C., Owen J.J.T., and Jenkinson E.J. (1996). Cellular interactions in thymocyte development. Annu. Rev. Immunol. **14**: 73-99.

Arnaout M.A. (1990). Structure and function of the leukocyte adhesion molecules CD11/CD18. Blood **75**: 1037-1050.

Berrih S.F., Arenzana-Seisdedos F., Cohen S., Devos R., Charron D., and Virelizier J. (1985). Interferon-gamma modulates HLA class II antigen expression on cultured thymic epithelial cells. J. Immunol. **135**: 1165-1171.

Boyd L.R., Tucek C.L., Godfrey D.I., Izon D.J., Wilson T.J., Davidson N.J. Bean A.G.D., Ladyman H.M., Ritter M.A., and

Hugo P. (1993). The thymic microenvironment. Inside the thymus. Immunol. Today **14**: 445-459.

Čolić M., Pejnović N., Kataranovski M., Stojanović N., Terzić T., and Dujić A. (1991). Rat thymic epithelial cells in culture constitutively secrete IL-1 and IL-6. Int. Immunol. **3**: 1165-1174.

Čolić M., Stojanović N., Popović Lj., and Dujić A. (1992). Phenotypic and ultrastructural characterization of an epithelial cell line established from rat thymic cultures. Immunology **77**: 201-207.

Čolić M., Vučević D., Miyasaka M., Tamatani T., Pavlović M.D., and Dujić A. (1994a). Adhesion molecules involved in the binding and subsequent engulfment of thymocytes by a rat thymic epithelial cell line. Immunology **83**: 449-456.

Čolić M., Vučević D., Stojanović N., Lukić T., Gašić S., Popović Lj., Pavlović M.D., Pejnović N., and Dujić A. (1994b). Similarities and differences in phenotypical and functional characteristics of cloned thymic epithelial cell lines. Vojnosanit. Pregled **51(4)**: 9-12.

Hiramine C., Hojo K., Koseto M., Nakagawa T., and Mukasa A. (1990). Establishment of a murine thymic epithelial cell line capable of inducing both thymic nurse cell formation and thymocyte apoptosis. Lab. Invest. **62**: 41-64.

Itoh T., Doi H., Chin S., Nishimura T., and Kasahara S. (1988). Establishment of mouse thymic nurse cell clones from a spontaneous BALB/c thymic tumor. Eur. J. Immunol. **18**: 821-824.

Le P.T., Vollger L.W., Haynes B.F., and Singer K.H. (1990). Ligand binding to the LFA-3 cell adhesion molecule induces IL1 production by human thymic epithelial cells. J. Immunol. **144**: 4541-4547.

Mc Cormic F. (1993). How receptors turn ras on. Nature **363**: 15-16.

Murray R., Suda T., Wrighton N., Lee F., and Zlotnik A. (1989). IL-7 is a growth and maintenance factor for mature and immature thymocyte subsets. Int. Immunol. **1**: 526-531.

Nakashima M., Mori K., Maeda K., Kishi H., Hirata K., Kawabuchi M., and Watanabe T. (1990). Selective elimination of double-positive immature thymocytes by a thymic epithelial cell line. Eur. J. Immunol. **20**: 47-53.

Popović Lj., Čolić M., and Kosec D. (1994). Production and characterization of the rat thymic T-cell hybridomas. Vojnosanit. Pregl. **51**: 56-59.

Popović P., Čolić M., and Vučević D. (In press). A medullary rat thymic epithelial cell line induces apoptosis of the BWRT 8 thymocyte hybridoma. Yugoslav Physiol. Pharmacol. Acta.

Potworowski F.E., Hugo P., and Couture C. (1989). Binding of cortical thymocytes to a medullary epithelial cell line: A brief review. Thymus **13**: 237-243.

Ray R.J., Furlonger C., Williams D.E., and Paige C.J. (1996). Characterization of thymic stromal-derived lymphopoietin (TSLP) in murine B cell development in vitro. Eur. J. Immunol. **26**: 10-16.

Ritter M.A., and Boyd R.L. (1993). Development in the thymus: It takes two to tango. Immunol. Today **14**: 462-469.

Schaller M.D., and Parsons J.T. (1993). Focal adhesion kinase: An integrin-linked protein tyrosine kinase. Trends Cell Biol. **3**: 258-262.

Suda T., Murray R., Guidas C., and Zlotnik A. (1990). Growth-promoting activity of IL-1 alpha, IL-6 and tumor necrosis factor-alpha in combination with IL-2, IL-4 or IL-7 on murine thymocytes. Differential effect on CD4/CD8 subsets and on CD3+/CD3-double-negative thymocytes. J. Immunol. **144**: 3039-3045.

Van Ewijk W. (1988). Cell surface topography of thymic microenvironments. Lab. Invest. **59**: 579-590.

Ward S.G., June C.H., and Olive D. (1996). PI 3-kinase: A pivotal pathway in T-cell activation? Immunol. Today. **17**: 187-197.

Wekerle H., and Ketelsen U.P. (1980). Thymic nurse cells: I-A bearing epithelium involved in T lymphocyte differentiation. Nature **283**: 402-404.

